# A Boy Who Knows No Pain: Anaesthetic Management of Congenital Insensitivity to Pain With Anhidrosis

**DOI:** 10.7759/cureus.30790

**Published:** 2022-10-28

**Authors:** Maria Paul, Charu Bamba, Vanya Chugh, Nisha Ravikumar, Jayaram S

**Affiliations:** 1 Anesthesia and Critical Care, Vardhman Mahavir Medical College and Safdarjung Hospital, New Delhi, IND

**Keywords:** cipa, analgesia, anaesthetic complications, perioperative management, autonomic dysregulation

## Abstract

Congenital insensitivity to pain with anhidrosis (CIPA) is a rare disorder with an absence of pain perception, anhidrosis, heat intolerance, and varying degrees of mental retardation. Though cases of CIPA have innate analgesia, they have been known to have tactile hyperesthesia, thus making anesthesia necessary in case of any surgery. Perioperative complications due to abnormal autonomic functions like bradycardia, hypotension, and hyperthermia are major challenges in the anesthetic management of these cases. Here, we report a case on the anesthetic management of CIPA.

## Introduction

Congenital insensitivity to pain with anhidrosis (CIPA) or hereditary sensory and autonomic neuropathy type IV is a rare autosomal recessive disorder with an incidence of 1 in 125 million newborns [1]. It is a condition caused by a mutation in the neurotrophic tyrosine receptor kinase 1 (NTRK1) gene located on chromosome 1 [2]. Symptoms include recurrent episodes of fever, absence of pain sensitivity, unconscious self-mutilation, developmental delay, etc. [3]. It has been noticed that it also affects the immune system causing slow healing and chronic inflammation [2]. The diagnosis is made based on symptoms, signs, pharmacological tests, nerve conduction velocity study, histopathologic examination of the skin, and genetic study revealing NTRK1 mutation [4]. The lack of pain alters the quality of life as patients need to check for injuries frequently. Cases of CIPA often present to the hospital with orthopedic symptoms from repeated trauma resulting in Charcot joints and osteomyelitis, at times requiring surgical intervention. There is no definitive method of anesthesia in CIPA, with records available of patients being operated on even without anesthesia [5]. The aim of reporting this case is to increase awareness about CIPA and the anesthetic management of such patients.

## Case presentation

**Figure 1:**
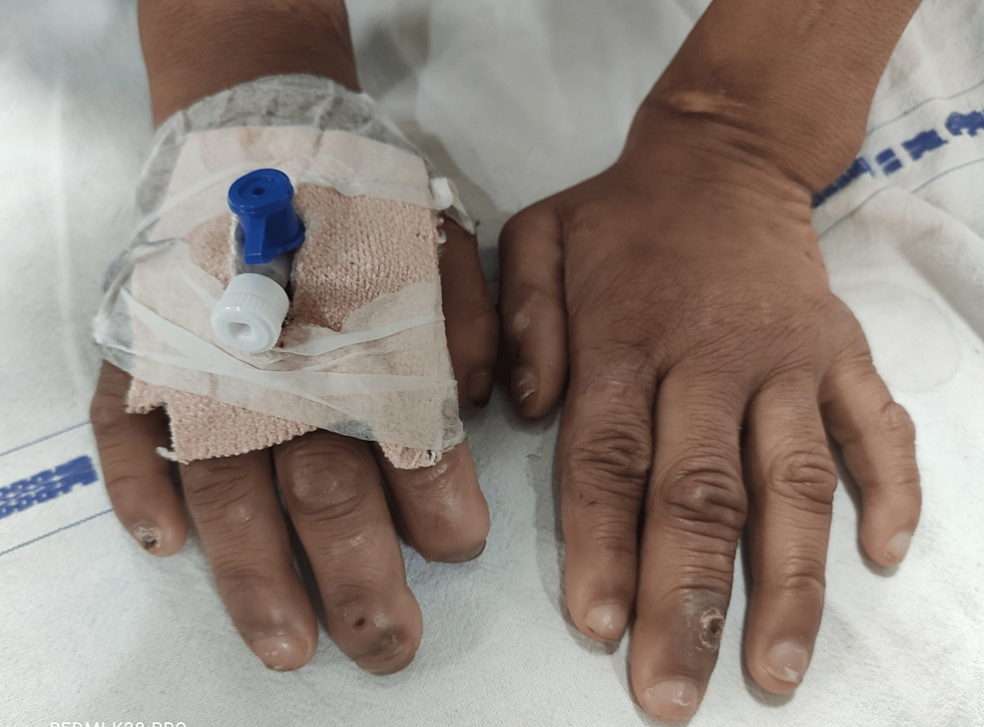
Both hands revealing acromutilation

**Figure 2:**
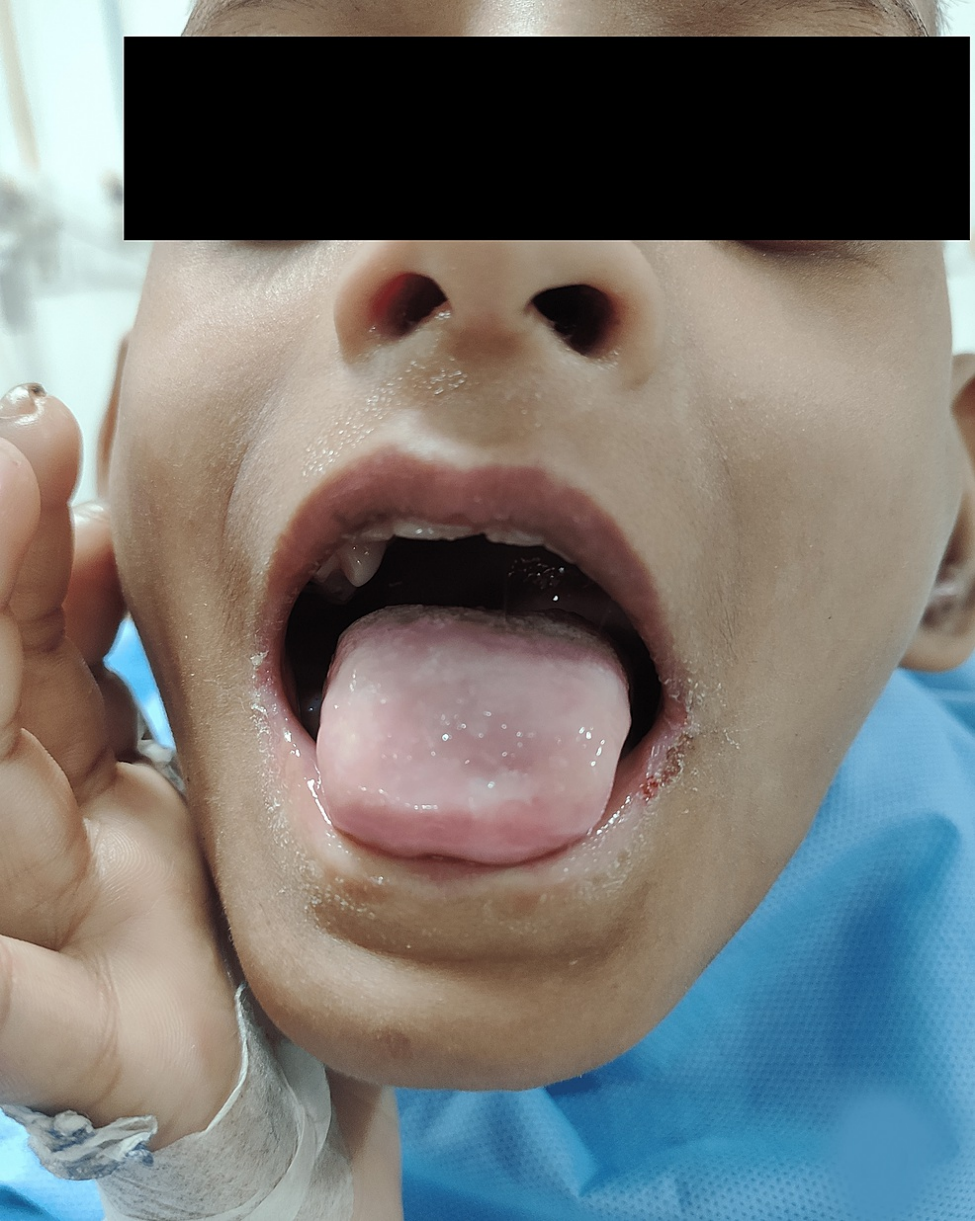
Peri-oral self-inflicted injuries

**Figure 3:**
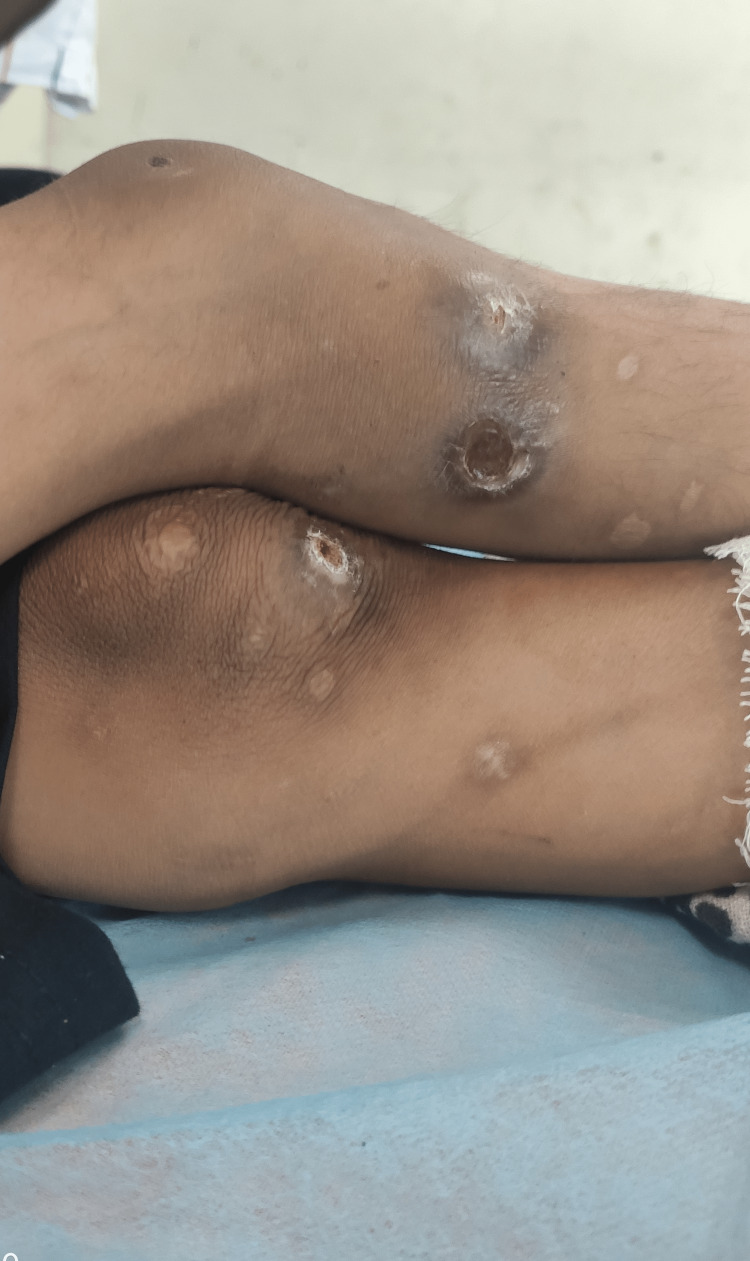
Multiple skin lesions over the leg due to injuries and osteomyelitis

**Figure 4:**
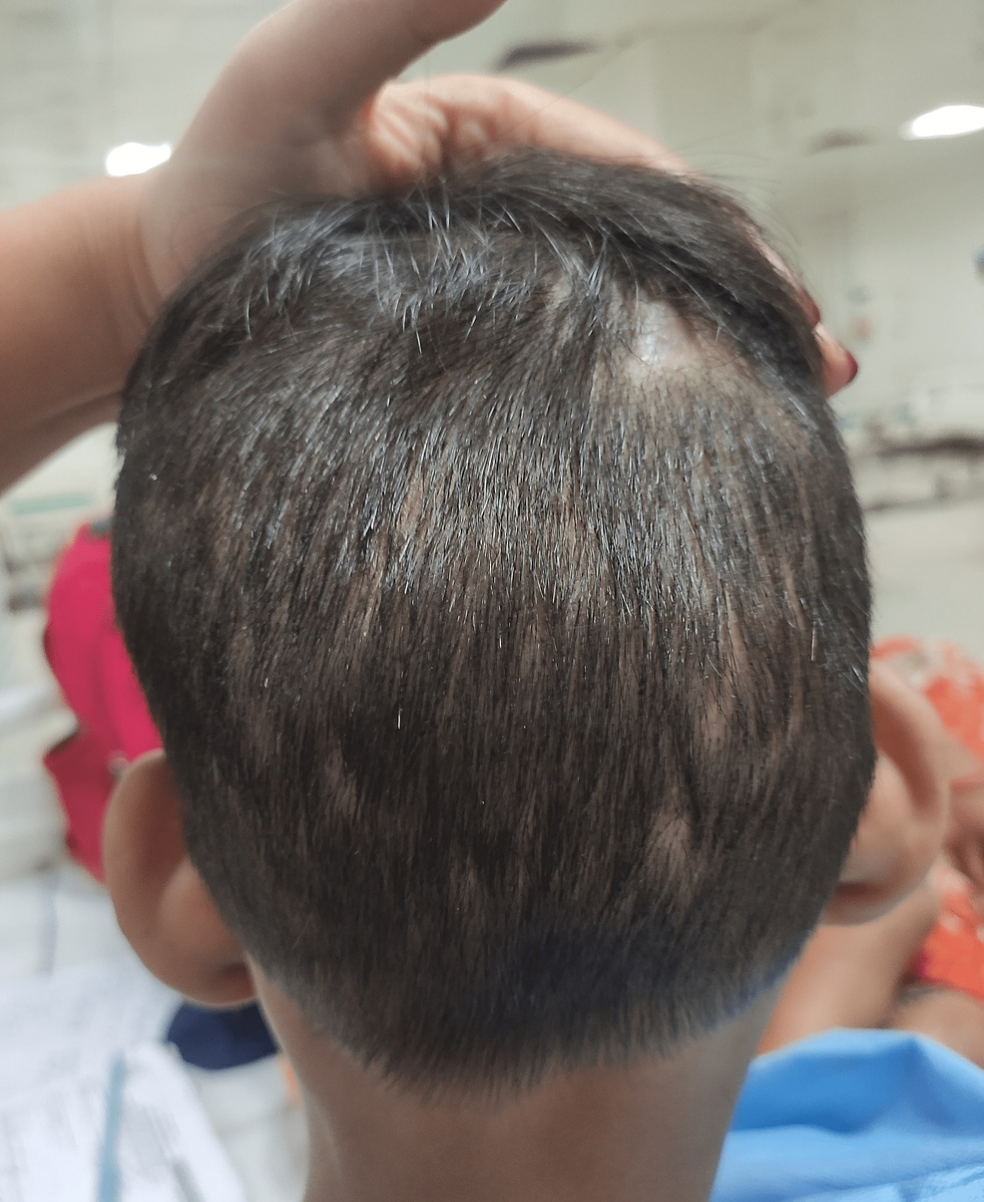
Patient with patchy hair loss

A seven-year-old boy (ASA II as per American Society of Anesthesiologists (ASA) physical status classification) of Indian ethnicity weighing 25.5 kg was planned to undergo incision and drainage for septic arthritis of the left ankle joint. A detailed pre-anesthetic evaluation revealed a history of episodes of fever, self-mutilation, multiple traumatic injuries in the past, and delayed developmental milestones. The inability of the child to sweat was revealed during the medical evaluation of repeated fevers in his infancy. He is an intellectually challenged child born out of a non-consanguineous marriage with no similar history in the family. The parents were unaware of pain insensitivity, as they were not educated about the condition and its implications. Physical examination revealed hyperkeratosis of joints, acromutilation (Figure 1), perioral self-mutilated injuries (Figure 2), multiple wounds and scars (Figure 3), and areas of patchy hair loss (Figure 4) with no tenderness of the left ankle joint. The vital parameters, including body temperature, were within normal limits. There was complete pain insensitivity with the inability to differentiate between hot and cold sensations. Deep tendon reflexes and corneal reflexes were intact. The routine blood investigations revealed moderate anemia (Hb: 8.9g/dl). A review of previous treatment records from another institute revealed that the child was diagnosed with CIPA after a skin biopsy, nerve conduction velocity study, autonomic function test, and genetic testing.

The management plan for the child was arthrotomy under general anesthesia with nil per oral (NPO) for six hours. After informed consent from parents, the child was taken to the operating room and placed on a padded operation table. The pre-induction vitals included heart rate - 102/min, respiratory rate - 16 cycles/min, and blood pressure - 89/62 mmHg. Intravenous (IV) access was secured. Monitoring of heart rate, non-invasive blood pressure (NIBP), five-lead ECG, oxygen saturation (SpO2), end-tidal carbon dioxide (ETCO2), and body temperature was done. Baseline vital parameters were within the normal limit for his age. The child was not actively warmed. Anesthesia was induced with a fentanyl injection of 2 µg/kg IV and a propofol injection of 1.5 mg/kg IV, and a cisatracurium injection of 0.15 mg/kg IV was given for muscle relaxation. The airway was secured with cuffed endotracheal (ET) tube intubation. Anesthesia was maintained with an anesthetic agent (sevoflurane) and nitrous oxide. Bispectral index (BIS) and age-adjusted minimum alveolar concentration (MAC_age_) were monitored. MAC_age_ was maintained between 0.8-1 and BIS was maintained between 40-60. After the 20-minute-long operation, volatile inhalational agents were tapered off and he was extubated after reversal with injection neostigmine 50 µg/kg and injection glycopyrrolate 10 µg/kg. The child was monitored further in the postoperative care unit. The intraoperative and postoperative periods were uneventful without hyperthermia or other autonomic dysregulations. There was no requirement for postoperative analgesics. The patient was discharged postoperatively on the second day in stable condition.

## Discussion

CIPA is a rare disorder on which very limited literature is available on its anesthetic management and complications. There is no definitive agreement on the anesthetic management of CIPA cases. There are records of cases of CIPA operated without anesthesia as well [5]. Although the patients are insensitive to pain, it is necessary to alleviate the anxiety associated with surgeries, which may cause stress and possibly lead to hemodynamic variations. This, along with the risk of accidental fractures, may necessitate the use of sedatives or anxiolytic drugs in the preoperative period [4]. CIPA cases have been known to have tactile hyperesthesia, reemphasizing the need for anesthesia [6]. Autonomic nervous system abnormalities causing perioperative complications increase the risk of hypotension, bradycardia, hyperthermia, regurgitation, and aspiration, which should be anticipated and watched for [7]. The incidence of these complications is not well known [8]. In our case, we administered general anesthesia to the patient with IV fentanyl and IV induction agents and maintained the anesthesia on inhalational agents. There was no preference for any opioid, induction agent, or inhalational agent. There was no event of autonomic dysregulation or postoperative nausea and vomiting (PONV).

Monitoring and controlling the body temperature was crucial. It is recommended to maintain the core body temperature < 37ºC perioperatively as hyperpyrexia could be fatal in around 20% of the patients in the first three years of life. Normal body temperature can be maintained by controlling the environmental temperature. Our patient was normothermic preoperatively and remained so in the intra and postoperative periods, thus not requiring any active warming or cooling. Patients with CIPA are distinctive due to inherent analgesia. However, fentanyl was given to abolish the stress response that can occur due to airway manipulation. In spite of the lack of sensations of pain, patients with CIPA still have tactile sensations and respond to airway manipulations [7,8]. There was no requirement for postoperative analgesia.

## Conclusions

Anaesthetic management of CIPA poses numerous challenges. Management of autonomic dysfunction, regulation of body temperature, and prevention of aspiration should be included in the anesthetic goals. Airway reflexes remain intact despite analgesia and therefore need to be prevented. Postoperative analgesia is not usually necessary in patients with CIPA.
